# The relationship between dementia caregivers and quality of life in South Korean populations

**DOI:** 10.1097/MD.0000000000038605

**Published:** 2024-06-21

**Authors:** Sujin Lee, Jae Ho Chung

**Affiliations:** a Department of Neurology, International St. Mary’s Hospital, Catholic Kwandong University College of Medicine, Incheon, Republic of Korea; b Internal Medicine, International St. Mary’s Hospital, Catholic Kwandong University College of Medicine, Incheon, Republic of Korea.

**Keywords:** dementia, family caregivers, quality of life

## Abstract

The purpose of this study was to assess the relationship between quality of life and dementia caregivers. The 2019 Korean Community Health Survey participants were assessed using the Patient Health Questionnaire-9, subjective cognitive decline (SCD) and SCD-related functional limitation, and EuroQol 5-dimension (EQ-5D). Sociodemographic and psychosocial variables were evaluated and compared between participants with dementia caregivers (n = 37,614) and non-dementia caregivers (n = 140,518). The dementia caregivers group reported significantly higher rates of depression, SCD, SCD-related functional restriction, and mean EQ-5D compared to the non-dementia caregivers group (*P* < .001). After adjusting for multiple confoundings, the odds ratio (OR) for depression (Patient Health Questionnaire-9 ≥ 10), SCD, SCD-related functional limitation, and lowest quartile of the EQ-5D index scores in the dementia caregivers group were 1.43 (95% confidence interval [CI], 1.29–1.59), 1.30 (95% CI: 1.24–1.36), 1.26 (95% CI: 1.20–1.32), and 1.22 (95% CI: 1.16–1.29), respectively. Physical activity (OR: 1.47; 95% CI: 1.43–1.52), self-control (OR: 1.41; 95% CI: 1.35–1.47), daily activity (OR: 1.55; 95% CI: 1.50–1.60), pain (OR: 1.62; 95% CI: 1.58–1.67), and anxiety/depression (OR: 2.17; 95% CI: 2.10–2.24) were all more common among participants in the dementia caregivers group than in the non-dementia family caregivers group. Depression, SCD, and a lower quality of life are linked to dementia caregivers, especially if there is moderate to severe anxiety or depression.

## 1. Introduction

Dementia caregivers suffer from physical tension psychosocial stress, depression, and anxiety.^[1]^ Besides, dementia caregivers suffer from economic problems, fall behind in their social activities and job efficiency declines compared with non-dementia caregivers. This continuous and cumulative disaster impacts their lives and eventually puts dementia caregivers at great risk of experiencing reduced quality of life (QoL). As the QoL deteriorates for dementia caregivers, many dementia caregivers decided to place dementia patients in assisted-living facilities or nursing homes.^[2]^

It was no wonder that the QoL in dementia caregivers and improvement strategy in the dementia caregivers QoL has been an area of concern. Recent studies have shown that the dementia caregivers' QoL was lower than the QoL who care for persons with other diseases.^[3,4]^ Measuring the quality of care received by dementia caregivers might be helpful in finding potential barriers to QoL for dementia caregivers.

This is especially useful for stakeholders (policymakers, care providers, etc) if interventions or policies can improve QoL barriers. For example, these interventions could provide information about changes in the QoL in the COVID-19 pandemic. In fact, many dementia caregivers face increasing barriers during COVID-19, including social isolation, lack of home care services, and lack of informal support.^[5,6]^

QoL is an important instrument for health status measurements.^[7]^ QoL is associated with sociodemographic factors such as sex, age, residence,^[8]^ lifestyle factors,^[9]^ emotional diseases such as depression and anxiety. EuroQol 5-dimension (EQ-5D) is a QoL questionnaire that is valid for cross-sectional studies.

Dementia caregivers would have lower QoL than non-dementia caregivers in our study hypothesis. To our knowledge, there was no study has been published on the between dementia caregivers and QoL in the general Korean population. Understanding the QoL of people with dementia and their caregivers is vital for the accurate evaluation of health interventions for care providers.^[10]^ So, in our study, we investigate whether there is an association between dementia caregivers and QoL, using data from the 2019 Korea Community Health Survey (KCHS).

## 2. Methods

### 2.1. Study participants

Our study used the 2019 KCHS, a countrywide, survey that representative of the whole South Korean population, for this investigation. Every participant provided written informed permission. A 2-step sampling procedure was employed by KCHS to ensure that the sample unit was representative of the total South Korean population.^[11]^ A total of 177,882 participants ranged in age from 40 to 108 years old, after excluding those with missing variables of subjective cognitive decline (SCD), Patient Health Questionnaire-9 (PHQ-9), and QoL measurements. The data from the KCHS are made publicly available. Before being released, all participant data were completely anonymized. Because the data were exempt from IRB review, our work was not included in the Enforcement Rule of Bioethics and Safety Act review list in Korea.

### 2.2. Sociographic variables

Age, sex, smoking, drinking alcohol, exercising, being married, working, having a family income, living area, educational attainment, stress level, health, SCD, depression as determined by the PHQ-9, and the presence of hypertension and diabetes mellitus were the sociographic variables in our study. A lifetime of >100 cigarettes was considered smoking.^[12]^ There were 2 categories for marriage status: married and not married. There were 2 categories for employment status: employed (full-time or part-time work) and unemployed. The classification of household income level was either above or below the average. Living quarters were divided into urban and rural categories. The 3 categories for self-rated health state were good (very good, good), moderate, and terrible (bad or very bad). Our study looked into the depression variable for altering QoL because depression was linked to QoL. To characterize depression, the PHQ-9 was utilized. The PHQ-9 score in Korea has been found to have reliable sensitivity and specificity for depression when it is >10 points.^[13]^ Based on this, we classified the participants in this study as either depression (PHQ-9 ≥ 10) or non-depression (PHQ-9 < 10). Dementia caregivers were assessed by asking the following question “Do you have family members (Parent In-Laws, Spouse, Sibling, Yourself, and Children) If one of your family members has dementia, who is it?.” We categorized as dementia caregivers group if the “yes,” if not we categorized as non-dementia caregivers group.

### 2.3. SCD

SCD and SCD-related functional impairments were investigated using the cognitive decline module of the Behavioral Risk Factor Surveillance System.^[14]^ “Have you experienced confusion or memory loss that is happening more often or is getting worse in the last year?” was the question used to characterize SCD. SCD might be a useful approach for identifying preclinical dementia.^[15]^ The study was divided into 2 groups: the control group (infrequently or never) and the SCD-related functional limitation group (always, usually, or occasionally).

### 2.4. QoL measures

The EuroQol (EQ)-5D questionnaire was used to define QoL. A self-rated method for assessing life quality is the EQ-5D. The 5 components that make up the EQ-5D are mobility; self-care; regular activities; pain/discomfort; and anxiety/depressive mood. QoL, a preference-based health status index, was evaluated by calculating the average scores of the EQ-5D index.^[16,17]^

### 2.5. Data analysis

The overall features of dementia caregivers and non-caregivers were compared using a chi-square test with complicated sampling and Rao–Scott correction to reflect the full population, as this study was meant to employ weighted data. Multiple logistic regression analysis with complex sampling was performed, adjusting for multiple confounding variables. All findings in this study are shown as weighted values. All statistical analyses and graphics were created using R programming language and a variety of tools. *P* < .005 was considered to indicate statistical significance.

## 3. Results

Of the 177,835 respondents, 10,634 (6.8%) reported that they were dementia caregivers group. The participants’ characteristics are reported in Table 1. Participants in the dementia caregivers group were younger, had lower education, were unmarried, lived in an urban location, and had fewer comorbid conditions (diabetes and hypertension) compared to the non-dementia caregivers group (*P* < .001). Table 2 displays the relationships between dementia caregiver status, psychological health, and QoL measures. Dementia caregivers reported higher stress levels than non-dementia caregivers (*P* < .001). Dementia caregivers reported considerably higher rates of depression (PHQ-9 scores ≥ 10) compared to non-dementia caregivers (*P* < .001). SCD (20,8% vs 24.6%) and SCD-related functional limitation (20.7% vs 24.0%) were higher in dementia caregivers than non-dementia caregivers (all *P* < .001). The mean EQ-5D index score was significantly lower in the dementia caregivers group than in the non-dementia caregivers group. The mean EQ-5D index score was 0.88 ± 0.13 in those in the dementia caregivers group and 0.89 ± 0.12 in those in the non-dementia caregivers group (*P* = .007). The dementia caregivers group also included a higher proportion of participants who reported some or severe problems in all 5 dimensions of the EQ-5D than the non-dementia caregivers group did. Table 3 shows the differences in psychological health and QoL variables between participants in the dementia caregivers group. After adjusting for multiple confounding variables, the odds ratio (OR) for depression (PHQ-9 ≥ 10), SCD, SCD-related functional limitation, and lowest quartile of the EQ-5D index scores in the dementia caregivers group were 1.43 (95% confidence interval [CI], 1.29–1.59), 1.30 (95% CI: 1.24–1.36), 1.26 (95% CI: 1.20–1.32), and 1.22 (95% CI: 1.16–1.29), respectively. Participants in the dementia caregivers group were more likely to report physical activity (OR: 1.10; 95% CI: 1.04–1.18), self-control (OR: 1.21; 95% CI: 1.11–1.32), daily activity (OR: 1.20; 95% CI: 1.12–1.28), pain (OR: 1.18; 95% CI: 1.13–1.23), and anxiety/depression (OR: 1.31; 95% CI: 1.23–1.39), respectively.

**Table 1:** General characteristic of participants.

	No dementia caregiver (n = 167201)	Dementia caregiver (n = 10634)	*P* value
Age, yr ± SD	58.1 ± 12.1	57.4 ± 11.1	<.001†
Sex, n (%*)			<.001‡
Male	73,574 (48.4)	4766 (47.6)	
Female	93,627 (51.6)	5868 (52.4)	
Job, n (%*)			<.001‡
Employed (partly or full-time)	101,534 (62.3)	6702 (64.1)	
Unemployed	65,667 (37.7)	3932 (35.9)	
Education, n (%*)			<.001‡
Less than high school	39,887 (35.0)	3122 (39.6)	
College or higher	127,314 (65.0)	7512 (60.4)	
Marital status, n (%*)			<.001‡
Current marital	122,015 (76.6)	2037 (82.3)	
Others	45,186 (23.4)	8597 (17.7)	
Residence, n (%*)			<.001‡
Rural	80,947 (20.9)	4787 (19.7)	
Urban	86,254 (79.1)	5847 (80.3)	
Family income, n (%*)			<.001‡
Less than average	33,853 (19.1)	1894 (16.0)	
More than average	133,348 (80.9)	8740 (84.0)	
Smoking, n (%*)	61,679 (40.3)	4024 (40.0)	<.001‡
Alcohol, n (%*)	60,244 (41.8)	4071 (42.3)	<.001‡
Exercise, n (%*)	65,503 (45.2)	4523 (47.3)	<.001‡
Hypertension, n (%*)	60,262 (20.0)	3489 (28.9)	<.001‡
Diabetes, n (%*)	24,356 (12.5)	1416 (11.5)	<.001‡

* Estimated mean or rate-adjusted recommended weighted value.

† Linear regression analysis with complex sampling, significance at *P* < .05.

‡ Chi-square test with Rao–Scott correction, significance at *P* < .05.

**Table 2:** Comparison of the psychological health, EQ-5 Dimensions, and EQ-5D index according to dementia caregiver.

	No dementia caregiver(n = 167201)	Dementia caregiver(n = 10634)	*P* value
Perceived stress, n (%*)			<.001‡
No	134,114 (78.2)	7932 (72.8)	
Yes	33,087 (21.8)	2702 (27.2)	
Sleep duration, n (%*)			<.001‡
≤5 h	32,319 (18.9)	2108 (19.9)	
6 h	47,861 (31.1)	3091 (30.6)	
7 h	50,441 (31.1)	3144 (30.6)	
8 h	29,836 (16.0)	1862 (15.7)	
≥9 h	6144 (2.9)	429 (3.2)	
Perceived health status, n (%*)			<.001‡
Good	48,913 (32.5)	3078 (31.1)	
Moderate	75,977 (48.2)	4904 (48.8)	
Bad	42,311 (19.3)	2652 (20.1)	
PHQ-9 score, n (%*)			<.001‡
Normal (0–4)	142,747 (85.9)	8680 (81.7)	
Mild (5–9)	19,145 (11.1)	1468 (14.1)	
Moderate (10–14)	3585 (2.1)	311 (2.8)	
Moderately severe (15–19)	1188 (0.6)	127 (1.0)	
Severe (20–27)	536 (0.3)	48 (0.4)	
Depression (PHQ-9 ≥ 10), n (%*)	5309 (6.3)	486 (8.5)	<.001‡
SCD, n (%*)	34,741 (19.0)	2614 (22.6)	<.001‡
SCD-related factor, n (%*)	34,528 (18.9)	2551 (22.3)	<.001‡
Depressed mood (2 wk in a row, %), n (%*)	10,259 (3.0)	901 (4.3)	<.001‡
EQ-5D index score, mean	0.90 ± 0.10	0.89 ± 0.12	<.001‡
EQ-5D, n (%*)			
Problem with mobility			<.001‡
No problem	132,156 (85.6)	8590 (84.8)	
Some problems	33,950 (14.0)	1956 (14.5)	
Severe problems	1095 (0.4)	88 (0.7)	
Problem with self-care			<.001‡
No problem	155,010 (95.1)	9825 (94.5)	
Some problems	11,002 (4.4)	699 (4.7)	
Severe problems	1189 (0.5)	110 (0.8)	
Problem with usual activity			<.001‡
No problem	142,321 (90.1)	9074 (89.5)	
Some problems	23,116 (9.2)	1398 (9.3)	
Severe problems	1764 (0.7)	162 (1.1)	
Problem with pain/discomfort			<.001‡
No problem	103,855 (66.7)	6450 (64.1)	
Some problems	56,809 (30.4)	3754 (32.4)	
Severe problems	6537 (2.9)	430 (3.6)	
Problem with anxiety/depression			<.001‡
No problem	142,758 (85.4)	8665 (82.1)	
Some problems	22,705 (13.7)	1804 (16.4)	
Severe problems	1738 (0.9)	165 (1.5)	

EQ-5D = EuroQol 5-dimension, PHQ-9 = Patient Health Questionnaire-9, SCD = subjective cognitive decline.

* Estimated mean or rate-adjusted recommended weighted value.

† Linear regression analysis with complex sampling, significance at *P* < .05.

‡ Chi-square test with Rao–Scott correction, significance at *P* < .05.

**Table 3:** Adjusted odds ratio of dementia caregiver for depression, subjective cognitive decline (SCD), SCD-related functional limitation, and some or severe problems in EQ-5D dimensions.

	OR (95% CI)
Depression (PHQ-9 ≥ 10)	1.43 (1.29–1.59)
SCD	1.30 (1.24–1.36)
SCD-related functional limitations	1.26 (1.20–1.32)
EQ-5D (lowest quartile, %)	1.22 (1.16–1.29)
EQ-5D	
EQ-1 (some or severe): physical activity	1.10 (1.04–1.18)
EQ-2 (some or severe): self-control	1.21 (1.11–1.32)
EQ-3 (some or severe): daily activity	1.20 (1.12–1.28)
EQ-4 (some or severe): pain	1.18 (1.13–1.23)
EQ-5 (some or severe): anxiety/depression	1.31 (1.23–1.39)

Adjusted for age, sex, area of residence, educational status, marital status, occupation, family income, self-rated health status, stress level, smoking status, alcohol intake, sleep duration, and presence of diabetes and hypertension.

CI = confidence interval, EQ-5D = EuroQol 5-dimension, OR = odds ratio, PHQ-9 = Patient Health Questionnaire-9, SCD = subjective cognitive decline.

## 4. Discussion

Our study showed that dementia caregivers were associated with depression, SCD, and impaired QoL, particularly if some or severe problems with anxiety/depression are present.

Our study showed that QoL assessed by EQ-5D was significantly impaired in dementia caregivers consistent with other studies.^[18,19]^ However, inconsistent study results found there were poor associations between dementia caregiver and dementia patient QoL^[20,21]^ which were assessed using the EQ-5D questionnaires. Our study findings might imply that the EQ-5D may be the most appropriate measure of the impact of caring for people with dementia caregivers. This suggests that, although the EQ-5D is commonly used in health economics and outcomes research involving caregivers of people with dementia.^[22]^ Thus, the EQ-5D may cost-effective method of intervention for dementia caregivers. QoL was an important strategy for healthcare providers (policymakers, care providers) because QoL not only reflects physical health or disease status but also social well-being and spiritual wellness status.^[23]^

Our study showed that depression assessed by PHQ-9 was significantly higher in dementia caregivers than in non-dementia caregivers consistent with the China study.^[24]^ The rate of depression assessed by PHQ-9 among dementia caregivers in our study was 24.6%. In Korea, there is a unique culture in which the eldest son takes care of his parents.^[25]^ Consequently, this culture is very stressful for married women to take care of their older parents as well as balance child-rearing activities and professional careers.^[26]^ This high prevalence of dementia caregiver depression might be needed to focus sufficient attention on the mental health of dementia caregivers and provide psychological support and interventions.

Our study showed that SCD and SCD-related functional limitations were significantly higher in dementia caregivers than in non-dementia caregivers compatible with other studies.^[27–31]^ Dementia caregivers were shown to have poorer objective cognitive function than non-dementia caregivers.^[27]^ Relative to non-dementia caregivers, dementia caregivers have more deficits in Memory tasks such as long-delay recall^[28]^ and spatial working memory impairments.^[29]^ These repetitive and severe stress increases the substantial risk for the development of dementia in dementia caregivers.^[30]^ Dementia caregivers also perform worse executive function than non-dementia caregivers which is especially important because it predicts the long-term ability to perform activities of daily living.^[31]^

Although there are various studies, emotional or physical conditions of dementia caregivers affect the QoL of the dementia caregivers.^[32]^ The 5 subcomponents of QoL (mobility, self-care, usual activities, pain/discomfort, and anxiety/depressive mood) score difference seen in our findings suggests that anxiety/depression subcomponents were the most significant difference. This is an adjustable risk factor, and assistance from dementia family members is essential in lowering the burden of dementia caregivers.

There are some limitations to our investigation. First off, this study does not provide data on the degree or duration of dementia. Second, while the PHQ-9 is a valuable tool for identifying depression, the participants’ subjective and imprecise descriptions of their depressive symptoms may be present. Third, there was no way to account for other psychological conditions among dementia caregivers. Fourth, since the design of our study was cross-sectional, it was not possible to determine a cause-and-effect link. Despite these drawbacks, our study has certain advantages. We employed the most recent national-wide datasets, which are representative databases, to investigate correlations between dementia caregivers and QoL. In addition, we used the EQ-5D, a simple and reliable measure for screening patients with QoL. To our knowledge, this is the first Korean study to use the EQ-5D with KCHS data to investigate the relationship between living with dementia patients and family caregivers’ QoL. In conclusion, we found that dementia caregivers are associated with depression, SCD, and impaired QoL. Among them, anxiety/depression was the most important subcomponent of QoL. More prospective studies might clarify the association between dementia caregivers and QoL and eventually might be helpful in establishing effective interventions for improving QoL in dementia caregivers.

## Author contributions

**Conceptualization:** Sujin Lee, Jae Ho Chung.

## References

[1] Bruvik FK Ulstein ID Ranhoff AH Engedal K. The quality of life of people with dementia and their family carers. Dement Geriatr Cogn Disord. 2012;34:7–14. 22854507 10.1159/000341584

[2] Scholzel-Dorenbos CJ Meeuwsen EJ Olde Rikkert MG. Integrating unmet needs into dementia health-related quality of life research and care: introduction of the hierarchy model of needs in dementia. Aging Ment Health. 2010;14:113–9. 20155528 10.1080/13607860903046495

[3] Hazzan AA Shannon H Ploeg J Raina P Gitlin LN Oremus M. The association between caregiver well-being and care provided to persons with Alzheimer’s disease and related disorders. BMC Res Notes. 2016;9:344. 27430976 10.1186/s13104-016-2150-z PMC4950605

[4] Hazzan AA Ploeg J Shannon H Raina P Oremus M. Association between caregiver quality of life and the care provided to persons with Alzheimer’s disease: protocol for a systematic review. Syst Rev. 2013;2:17. 23497507 10.1186/2046-4053-2-17 PMC3610300

[5] Greenberg NE Wallick A Brown LM. Impact of COVID-19 pandemic restrictions on community-dwelling caregivers and persons with dementia. Psychol Trauma. 2020;12:S220–1. 32584105 10.1037/tra0000793

[6] Cohen G Russo MJ Campos JA Allegri RF. Living with dementia: increased level of caregiver stress in times of COVID-19. Int Psychogeriatr. 2020;32:1377–81. 32729446 10.1017/S1041610220001593 PMC7453351

[7] Guyatt GH Veldhuyzen Van Zanten SJ Feeny DH Patrick DL. Measuring quality of life in clinical trials: a taxonomy and review. CMAJ. 1989;140:1441–8. 2655856 PMC1269981

[8] Zhou Z Zhou Z Gao J Lai S Chen G. Urban-rural difference in the associations between living arrangements and the health-related quality of life (HRQOL) of the elderly in China-Evidence from Shaanxi province. PLoS One. 2018;13:e0204118. 30235258 10.1371/journal.pone.0204118 PMC6147447

[9] Eaglehouse YL Schafer GL Arena VC Kramer MK Miller RG Kriska AM. Impact of a community-based lifestyle intervention program on health-related quality of life. Qual Life Res. 2016;25:1903–12. 26896960 10.1007/s11136-016-1240-7 PMC5496447

[10] Chertkow H Feldman HH Jacova C Massoud F. Definitions of dementia and predementia states in Alzheimer’s disease and vascular cognitive impairment: consensus from the Canadian conference on diagnosis of dementia. Alzheimer’s Res Ther. 2013;5(Suppl 1):S2. 24565215 10.1186/alzrt198 PMC3981054

[11] Rim H Kim H Lee K. Validity of self-reported healthcare utilization data in the Community Health Survey in Korea. J Korean Med Sci. 2011;26:1409–14. 22065895 10.3346/jkms.2011.26.11.1409 PMC3207042

[12] Health UCfDCaP, behaviors of adults: United States V, and Health Statistics S, Number 245, Appendix II, 80

[13] Han C Jo SA Kwak JH. Validation of the Patient Health Questionnaire-9 Korean version in the elderly population: the Ansan Geriatric study. Compr Psychiatry. 2008;49:218–23. 18243897 10.1016/j.comppsych.2007.08.006

[14] Taylor CA Bouldin ED McGuire LC. Subjective cognitive decline among adults aged ≥45 years - United States, 2015-2016. MMWR Morb Mortal Wkly Rep. 2018;67:753–7. 30001562 10.15585/mmwr.mm6727a1 PMC6047468

[15] Jessen F Amariglio RE van Boxtel M. A conceptual framework for research on subjective cognitive decline in preclinical Alzheimer’s disease. Alzheimer's. 2014;10:844–52. 10.1016/j.jalz.2014.01.001 PMC4317324 24798886

[16] Kim SH Ahn J Ock M. The EQ-5D-5L valuation study in Korea. Qual Life Res. 2016;25:1845–52. 26961008 10.1007/s11136-015-1205-2

[17] Lee YK Nam HS Chuang LH. South Korean time trade-off values for EQ-5D health states: modeling with observed values for 101 health states. Value Health. 2009;12:1187–93. 19659703 10.1111/j.1524-4733.2009.00579.x

[18] Reed C Barrett A Lebrec J. How useful is the EQ-5D in assessing the impact of caring for people with Alzheimer’s disease? Health Qual Life Outcomes. 2017;15:16. 28109287 10.1186/s12955-017-0591-2 PMC5251250

[19] Bleijlevens MH Stolt M Stephan A. Changes in caregiver burden and health-related quality of life of informal caregivers of older people with dementia: evidence from the European RightTimePlaceCare prospective cohort study. J Adv Nurs. 2015;71:1378–91. 25403434 10.1111/jan.12561

[20] Lopez-Bastida J Serrano-Aguilar P Perestelo-Perez L Oliva-Moreno J. Social-economic costs and quality of life of Alzheimer disease in the Canary Islands, Spain. Neurology. 2006;67:2186–91. 17190942 10.1212/01.wnl.0000249311.80411.93

[21] Mesterton J Wimo A By A Langworth S Winblad B Jönsson L. Cross sectional observational study on the societal costs of Alzheimer’s disease. Curr Alzheimer Res. 2010;7:358–67. 19939223 10.2174/156720510791162430

[22] Jones C Edwards RT Hounsome B. Health economics research into supporting carers of people with dementia: a systematic review of outcome measures. Health Qual Life Outcomes. 2012;10:142. 23181515 10.1186/1477-7525-10-142 PMC3541129

[23] Bakas T McLennon SM Carpenter JS. Systematic review of health-related quality of life models. Health Qual Life Outcomes. 2012;10:134. 23158687 10.1186/1477-7525-10-134 PMC3548743

[24] Cheng Y Wang Z Yang T Lv W Huang H Zhang Y. Factors influencing depression in primary caregivers of patients with dementia in China: a cross-sectional study. Geriatr Nurs. 2021;42:734–9. 33857837 10.1016/j.gerinurse.2021.03.017

[25] Kang SJ Choi-Kwon S. Quality of life in the patients with Alzheimer’s disease and their caregivers: caregivers’ point of view. Korean J Adult Nurs. 2016;28:367–77.

[26] Kang HS Go KJ Kim WO. The lives of daughters-in-law who care for parents with dementia. J Korean Acad Nurs. 1999;29:1233–43.

[27] Caswell LW Vitaliano PP Croyle KL Scanlan JM Zhang J Daruwala A. Negative associations of chronic stress and cognitive performance in older adult spouse caregivers. Exp Aging Res. 2003;29:303–18. 12775440 10.1080/03610730303721

[28] Vitaliano PP Zhang J Young HM Caswell LW Scanlan JM Echeverria D. Depressed mood mediates decline in cognitive processing speed in caregivers. Gerontologist. 2009;49:12–22. 19363000 10.1093/geront/gnp004 PMC2664616

[29] Mackenzie CS Smith MC Hasher L Leach L Behl P. Cognitive functioning under stress: evidence from informal caregivers of palliative patients. J Palliat Med. 2007;10:749–58. 17592987 10.1089/jpm.2006.0171 PMC1905830

[30] Norton MC Smith KR Østbye T. Greater risk of dementia when spouse has dementia? The Cache County study. J Am Geriatr Soc. 2010;58:895–900. 20722820 10.1111/j.1532-5415.2010.02806.x PMC2945313

[31] Herrera AP George R Angel JL Markides K Torres-Gil F. Variation in older Americans act caregiver service use, unmet hours of care, and independence among Hispanics, African Americans, and Whites. Home Health Care Serv Q. 2013;32:35–56. 23438508 10.1080/01621424.2012.755143 PMC3583214

[32] Lim J-w Zebrack B. Caring for family members with chronic physical illness: a critical review of caregiver literature. Health Qual Life Outcomes. 2004;2:50. 15377384 10.1186/1477-7525-2-50 PMC521496

